# Antimalarial Activity of Ultra-Short Peptides †

**DOI:** 10.3390/molecules14125103

**Published:** 2009-12-08

**Authors:** Lemuel Pérez-Picaso, Benjamín Velasco-Bejarano, A. Berenice Aguilar-Guadarrama, Rocío Argotte-Ramos, María Yolanda Rios

**Affiliations:** 1 Centro de Investigaciones Químicas, Universidad Autónoma del Estado de Morelos, Avenida Universidad 1001, Col. Chamilpa, 62209 Cuernavaca, Morelos, México; 2 Instituto Nacional de Salud Pública, Centro de Investigación Sobre Enfermedades Infecciosas (CISEI), Avenida Universidad 655, Col. Santa María Ahuacatitlán, 62100 Cuernavaca, Morelos, México

**Keywords:** tetrapeptides, antimalarial activity, *Plasmodium berghei*, ultra-short peptides

## Abstract

Ultra-short peptides **1**-**9** were designed and synthesized with phenylalanine, ornithine and proline amino acid residues and their effect on antimalarial activity was analyzed. On the basis of the IC_50_ data for these compounds, the effects of nature, polarity, and amino acid sequence on *Plasmodium berghei* schizont cultures were analyzed too. Tetrapeptides Phe-Orn-Phe-Orn (**4**) and Lys-Phe-Phe-Orn (**5**) showed a very important activity with IC_50_ values of 3.31 and 2.57 μM, respectively. These two tetrapeptides are candidates for subsequent *in vivo* assays and SARS investigations.

## 1. Introduction

Malaria is one of the most lethal and widespread infectious parasite diseases in the world, affecting approximately 300 million people annually, mainly from developing countries like Central- and South-America, Asia, and Sub-Saharan Africa [1,2]. According to the World Health Organization, the number of clinical cases has reached between 300 and 500 million people per year [3]. About 1.1-2.7 million people in tropical and subtropical regions die of malaria every year [1,4,5,6]. In humans, it is caused by protozoan parasites from four *Plasmodium* species: *P. falciparum*, *P. vivax*, *P. malariae* and *P. ovale*. The most serious infections among these species are caused by *P. falciparum* [7,8]. In recent years, the social impact of malaria has increased due, to the lower abundance and high cost of artemisinin and related products (their effective distribution to combat malaria in economically disadvantaged regions could require an annual global subsidy of $300-500 million) [9], and to the emergence of resistant strains of *P. falciparum* and *P. vivax* to chloroquine, mefloquine, pyrimethamine, and to immunoprophylactic methods (such as vaccines) [10]. For these reasons, there is an increasing need for new chemical pharmacophores which may prove effective therapeutic antimalarial agents.

Several antimalarial peptides have been isolated from natural sources or obtained synthetically [8,11,12,13,14,15,16,17,18,19,20,21]. Lineal antimalarial peptides with fifteen or more amino acid residues, like leukinostatin A, efrapeptin, zervamicin and antiamoebin have been isolated [22], however shorter peptides with potent biological activity have also been described, among them lineal di-, tri-, tetra- and pentapeptides and their cyclic analogs [23,24,25,26,27,28]. Several of these peptides showed important activity against *P. falciparum*: pepstatin A (IC_50_ = 7.5 μM), a pentapeptide including a statine amino acid that was isolated from the culture of several *Actinomyces* species [23]; cyclotetrapeptides HC-toxin I, apicidin and apicidin A (MIC = 31, 125 and 125 ng/mL, respectively) [24]; linear tetrapeptide hirsutellic A acid (IC_50_ = 8.0 μM), isolated from *Hirsutella sp*. BCC 1528 fermentation broth, possessing an anthranilic acid residue at the *C*-terminus [12]; dipeptides 2-(2-*tert*-butoxycarbonylamino-4-methylpentanoyl-amino)-cyclooctanecarboxylic acid methyl ester and 2-(2-*tert*-butoxycarbonylamino-3-phenyl-propionylamino)-cyclooctanecarboxylic acid methyl ester are very effective *in vitro* (IC_50_ = 3.87 and 3.64 μg/mL, respectively); both compounds have maximum activity on maturation schizonts and total inhibition growth assays probed by total growth inhibition instead of stopping the cell division cycle [25]. Recent studies have showed that some commercial antiviral dipeptides used for HIV treatment, among them saquinavir, atazanavir and BR314, inhibit *P. falciparum in vitro* [26]. Additionally, some potent plasmepsin II inhibitors (peptidomimetics based on phenylalanine-statine amino acids) and of cysteine proteins have been studied as potential antimalarial agents [27]; in addition peptidylfluoromethyl ketones are potent inhibitors of falcipain, and block the degradation of hemoglobin and the parasite development at nanomolar concentrations [28]; Z-Phe-Arg-CH_2_F is a very potent inhibitor of trophozoite cysteine proteinase (TCP), and morpholine urea (Mu)-Phe-HPh-CH_2_F has an IC_50_ = 0.003 μM (after four days of subcutaneous administration) against *P. falciparum*, and cured 80% of malarial infected mice [30,31]. Moreover, apicidin (isolated from *Fusarium pallidoroseum*) is a potent *in vivo* antimalarial agent against *P. berghei* [29], while 2-(2-*tert*-butoxycarbonylamino-3-phenyl-propionylamino)-cyclooctanecarboxylic acid methyl ester was also active *in vivo* against this protozoan parasite [25]. In the present study, we report the antimalarial activity of some ultra-short peptides designed with the purpose of analyzing the effect of phenylalanine, ornithine and proline amino acid residues present on antimalarial activity.

## 2. Results and Discussion

Cationic (including arginine, lysine, ornithine and histidine residues, for example) and bulky (like proline, phenylalanine, valine, isoleucine and tryptophan) side chains are essential for antimicrobial activity in peptides [32] because they can confer amphiphatic character (simultaneous lipophilicity and hydrophilicity properties). In this paper, we analyzed the influence of the amphiphatic character of ultra-short peptides (including four or less amino acids residues) on antimalarial activity.

The amino acids statine, alanine, tyrosine, arginine, leucine, isoleucine, valine, proline, phenylalanine and lysine are frequently included on antimicrobial and antimalarial peptides [11,12,13,14,15,16,17,18,19,20,21]. On the other hand, it has been established that peptides, including ornithine residue (non-proteinogenic amino acid), show enzymatic and protein degradation stability [33]. For this reason, we used isoleucine, valine, proline, phenylalanine, lysine and ornithine amino acids to synthesize the ultra-short peptides studied in the present work.

Peptides **1**-**9** were synthesized in solid phase using Fmoc strategy and were designed to investigate the nature, polarity, and amino acid sequence on *Plasmodium berghei* schizont cultures. Our main purpose was to explore their potential antimalarial activity. On this context, peptides **1**-**4** were prepared to investigate the effect of *N*-terminal amino acid, while sequences in compounds **3** and **5** allowed us to analyze the effect of a change on the second amino acid residue on the antimalarial activity. With this same purpose, antimalarial activity of compound **2** was compared with its retropeptide sequence at compound **6**. Tetrapeptides **1**-**5** were synthesized to study the activity of peptides including Xaa-Xbb-Phe-Orn sequence, while the tetrapeptides **7** and **8**, and compound **9** were prepared to analyze the influence of the presence of proline residue, and tripeptide nature on antimalarial activity, respectively. Compounds **1**-**9** were evaluated *in vitro* for chemosuppression during 16 h with *P. berghei* schizont cultures. The results of the biological assay against this parasite blood stage are indicated in Table 1. Peptide **1** showed moderate antimalarial activity, with an IC_50_ = 32.35 μM. Substitution of a valine residue for isoleucine leads to a more active compound (peptide **2**), showing an IC_50_ = 10.00 μM, suggesting that polarity of the amino acid at the *N*-terminal residue is important for the antimalarial activity. In this case, substitution of a hydrogen in valine for a methyl group (isoleucine) confers more lipophilicity to the peptide and results in diminished antimalarial activity. Probably, the chiral center in the isoleucine residue is another reason for this decreased activity. The retropeptide of compound **2** (peptide **6**) was inactive, indicating that amino acid sequence is very important for the activity.

Tetrapeptides **2** and **3** have similar activity (IC_50_ = 10.00 *vs*. 13.48 μM) values, however, the IC_50_ value was about three times lower when the *N*-terminal lysine residue was substituted by phenyl-alanine (compound **4**, IC_50_ = 3.31 μM), and almost five times lower when the second amino acid phenylalanine residue was replaced with ornithine (compound **5**, IC_50_ = 2.57 μM). 

IC_50_ data for compounds **4** and **5** corroborate that *N*-terminal residue nature is very important for antimalarial activity, and that when a polar residue (like lysine or ornithine) is substituted by a less polar residue (like phenylalanine) antimalarial activity is increased. In our hands, peptides possessing the proline residue (compounds **7** and **8**) and tripeptide **9** were inactive. These results are in accordance with the findings of other authors who have suggested that the presence of non polar amino acid residues (providing lipophilic anchors) dramatically increases the biological activity, because those residues might be helping their penetration (disruption) through plasma membrane [25,32].

## 3. Conclusions

Tetrapeptides **1**-**5** including the sequence Xaa-Xbb-Phe-Orn showed antimalarial activity during *in vitro* schizont development assays, which significantly increased when a second phenylalanine amino acid (aromatic residue) was present, such as in compounds **4** and **5**. These compounds showed the highest antimalarial activity, with IC_50_ = 3.31 and 2.57 µM, respectively, and will be candidates for subsequent *in vivo* assays. These results showed that compounds **4** and **5** include interesting amino acid sequences for future SAR investigations.

## 4. Experimental

### 4.1. General procedures

Melting points were obtained in a Fisher Johns melting point apparatus and are uncorrected. Infrared (IR) spectra were obtained in KBr on a Bruker Vector 22 IR spectrometer. Optical rotations were measured in MeOH with sodium light on a Perkin-Elmer 341 MC polarimeter. ^1^H- and ^13^C-NMR spectra were recorded on a Varian Unity 400 spectrometer operating at 400 MHz for ^1^H-NMR, ^1^H-^1^H COSY, HSQC, and HMBC, and at 100 MHz for ^13^C-NMR, using CD_3_OD as solvent. Chemical shifts are reported in ppm (δ) relative to the TMS signal. FAB^+^MS, and HRFAB^+^MS were recorded on a JEOL JMStation-JM 700 mass spectrometer at 6 eV in a matrix of glycerol. Flash column chromatography (FCC) and analytical thin-layer chromatography (TLC) were performed using silica gel 230-400 mesh and pre-coated silica gel 60 F254 Merck plates, respectively. Fmoc-Lys(Boc)-OH, Fmoc-Orn(Boc)-OH, Fmoc-Phe-OH, Fmoc-Val-OH, and HBTU were obtained from GENSCRIPT. Fmoc-Ile-OH, Fmoc-Pro-OH, and HOBt were obtained from ANASPEC, and they were all used without further purification.

### 4.2. Cell culture and antimalarial activity measurements

Chloroquine-sensitive cultured *Plasmodium berghei* schizonts (ANKA 2.34) were used to assess the antimalarial activity of compounds **1**-**9**. Mature *P. berghei* schizonts were prepared as described by Thathy and Menard [34]. Peptides **1**-**9** were dissolved in PBS containing DMSO 0.1% and 5% ethanol. Parasites were incubated for 16 h at 37 °C as described. Chloroquine (C6628 Sigma Chloroquine diphosphate salt solid, ≥98% purity) was used as positive control. Samples of 0.5 mL of each culture were taken to prepare smears and used to count numbers of schizonts in 2,000 erythrocytes. The IC_50_ values of each compound were determined using concentrations of 5, 50, 100, and 200 µM, according to Khalid *et al.* [35], and by extrapolation from the concentration response curve. The IC_50_ value represents the drug concentration producing a 50% reduction in the number of *P. berghei* schizonts (compared to drug-free control cultures). For each assay, each drug dilution was analyzed in duplicate, and the results were averaged in each case. Based on NMR spectroscopic and HPLC chromatographic analyses, all compounds were at least of 98% purity.

### 4.3. General synthesis procedure for tri- and tetrapeptides ***1-9***

Solid phase synthesis of the peptides **1**-**9** [Ile-Lys-Phe-Orn (**1**), Val-Lys-Phe-Orn (**2**), Lys-Orn-Phe-Orn (**3**), Phe-Orn-Phe-Orn (**4**), Lys-Phe-Phe-Orn (**5**), Orn-Phe-Lys-Val (**6**), Lys-Val-Pro-Orn (**7**), Lys-Val-Phe-Pro (**8**), Phe-Orn-Val (**9**)] was carried out manually on a 2-Cl-Trityl-Cl resin (1 g, 1%, 200-400 mesh) using standard Fmoc-strategy methodology. Side-chain protecting groups were Orn(Boc) and Lys(Boc). All protected amino acids were coupled using a 0.45 M solution of HBTU/HOBt in DMF (2.1 mL × 0.25 mmol Fmoc aminoacid) and DIEA (2 mmol × 0.25 mmol Fmoc aminoacid) until completion (30 min to 1 h), judged by the Kaiser ninhydrin test. After coupling of the appropriate amino acid, Fmoc deprotection was effected by use of 20% piperidine/DMF solution for 2 min first, and then repeated for 30 min, followed by washing with DMF four times (10 mL each). The synthetic cycle was repeated to assemble the resin-bond protected peptide. The peptides were cleaved from the resin with simultaneous side-chain deprotection by acidolysis with 3% TFA/CH_2_Cl_2_ (1:1, 2 × 10 mL) containing 5% anisole at rt for 1 h. Cold diethyl ether (10 mL) was added and the precipitated peptide was filtered through a fine sintered funnel. The crude peptides were subjected first to FCC, and then purified to chromatographic homogeneity in the range of 90 to 95% by reverse-phase high-pressure liquid chromatography, performed on a semipreparative Phenomenex Luna 5C18 column, 250 × 4.6 mm; mobile phase: 80% acetonitrile, 0.1% TFA; flow rate: 1.2 mL/min, 220 nm). The purified peptides were subjected to amino acid analysis in order to confirm their composition. Peptides were stored as a lyophilized powder at 220 °C. Prior to experimentation, fresh solutions were prepared in water, vortexed, sonicated, and centrifuged, and the supernatant was diluted in the appropriate medium. 

*Ile-Lys-Phe-Orn* (**1**). White solid; Mp 160-162 °C; R_f_ 0.30 (BuOH:AcOH:H_2_O, 2:4:1); [α] – 1.74 (c 0.69, MeOH); IR: 3,434, 2,936, 1,674, 1,534, 1,198, 1,139, 838, 799, 694, 603 cm^-1^; ^1^H-NMR: δ 7.31-7.15 (5H, m, Ar), 4.61 (1H, dd, *J* = 9.6, 5.2 Hz, Hα-Phe), 4.41 (2H, m, Hα-Lys, Hα-Orn), 3.74 (1H, d, *J* = 6.0 Hz, Hα-Ile), 3.18 (1H, dd, *J* = 14.0, 4.8 Hz, Hβ-Phe), 2.95 (5H, m, Hβ’-Phe, Hδ-Orn, Hε-Lys), 1.97 (1H, m, Hβ-Orn), 1.84 (1H, m, Hβ-Ile), 1.77 (5H, m, Hβ-Orn, Hγ-Orn, Hβ-Lys), 1.67 (2H, m, Hγ-Lys), 1.51 (1H, m, Hγ-Ile), 1.42 (2H, m, Hγ-Lys), 1.14 (1H, m, Hγ’-Ile), 0.91 (3H, t, *J* = 7.2 Hz, Hδ-Ile), 0.89 (3H, d, *J* = 6.8 Hz, Hβ’-Ile); ^13^C-NMR: δ 174.37 (s, CO-Phe), 173.59 (s, CO-Orn), 173.37 (s, CO-Lys), 169.39 (s, CO-Ile), 138.16 (s, Ar), 130.30 (d, Ar), 129.48 (d, Ar), 127.82 (d, Ar), 58.82 (d, Cα-Ile), 56.32 (d, Cα-Phe), 54.45 (d, Cα-Lys), 53.06 (d, Cα-Orn), 40.55 (t, Cδ-Orn), 40.29 (t, Cε-Lys), 38.78 (t, Cβ-Phe), 38.11 (d, Cβ-Ile), 32.74 (t, Cβ-Lys), 29.63 (t, Cβ-Orn), 28.16 (t, Cδ-Lys), 25.58 (t, Cγ-Ile), 25.12 (t, Cγ-Orn), 23.69 (t, Cγ-Lys), 15.28 (q, Cβ’-Ile), 11.78 (q, Cδ-Ile); FAB^+^MS *m/z* (% ra); 521 (49) [M + H]^+.^, 307 (14) [M + H - C_11_H_24_N_3_O]^+^, 289 (13) [M - C_11_H_24_N_3_O - NH_3_]^+^, 176 (23), 154 (100), 136 (84), 84 (62), 77 (30); HRFAB^+^MS: observed 521.3468 [M + H]^+^, (calcd. for C_26_H_45_N_6_O_5_, 521.3451).

*Val-Lys-Phe-Orn* (**2**). Colorless solid; Mp 125-127 °C; R_f_ 0.35 (BuOH:AcOH:H_2_O, 2:4:1); [α] – 2.07 (c 0.66, MeOH); IR: 3,433, 2,938, 1,675, 1,535, 1,440, 1,200, 1,141, 840, 800, 718, 603 cm^-1^; ^1^H- NMR: δ 7.30-7.16 (5H, m, Ar), 4.62 (1H, dd, *J* = 9.2, 4.8 Hz, Hα-Phe), 4.40 (2H, m, Hα-Lys, Hα-Orn), 3.70 (1H, d, *J* = 6.0 Hz, Hα-Val), 3.18 (1H, dd, *J* = 14.4, 5.2 Hz, Hβ-Phe), 2.94 (5H, m, Hβ’-Phe, Hδ-Orn, Hε-Lys), 2.11 (1H, dh, J = 6.8, 5.6 Hz, Hβ-Val), 1.97 (1H, m, Hβ-Orn), 1.77 (3H, m, Hβ’-Orn, Hβ-Lys), 1.66 (4H, m, Hγ-Orn, Hδ-Lys), 1.42 (2H, m, Hγ-Lys), 0.95 (6H, d, *J* = 6.8 Hz, Hγ-Val, Hγ’-Val); ^13^C-NMR: δ 174.35 (s, CO-Phe), 173.56 (s, CO-Orn), 173.43 (s, CO-Lys), 169.46 (s, CO-Val), 138.13 (s, Ar), 130.32 (d, Ar), 129.50 (d, Ar), 127.81 (d, Ar), 59.59 (d, Cα-Val), 56.27 (d, Cα-Phe), 54.54 (d, Cα-Orn), 53.04 (d, Cα-Lys), 40.53 (t, Cδ-Orn), 40.30 (t, Cε-Lys), 38.75 (t, Cβ-Phe), 32.73 (t, Cβ-Lys), 31.70 (t, Cβ-Val), 29.63 (t, Cβ-Orn), 28.16 (t, Cδ-Lys), 25.12 (t, Cγ-Orn), 23.71 (t, Cγ-Lys), 19.16 (q, Cγ-Val), 18.14 (q, Cγ’-Val); FAB^+^MS *m/z* (% ra): 507 (39) [M + H]^+^, 307 (14), 289 (10), 176 (14), 154 (100), 136 (65), 120 (38), 107 (29), 89 (34), 84 (65), 77 (31), 72 (86); HRFAB^+^MS: observed 507.3288 [M + H]^+^, (calcd. for C_25_H_43_N_6_O_5_, 507.3295).

*Lys-Orn-Phe-Orn* (**3**). White solid; Mp 123-125 ^o^C; R_f_ 0.20 (BuOH:AcOH:H_2_O, 2:4:1); [α] + 0.92 (c 2.01, MeOH); IR: 3,434, 3,072, 2,937, 1,675, 1,535, 1,439, 1,198, 1,139, 839, 800, 718, 603 cm^-1^; ^1^H- NMR: δ 7.32-7.16 (5H, m, Ar), 5.65 (1H, dd, *J* = 9.2, 5.2 Hz, Hα-Phe), 4.40 (2H, m, Hα of both Orn), 3.92 (1H, t, *J* = 6.4 Hz, Hα-Lys), 3.17 (1H, dd, *J* = 14.4, 5.2 Hz, Hβ-Phe), 2.93 (5H, m, Hβ’-Phe, Hδ-Orn, Hε-Lys), 1.96 (2H, m, Hβ of both Orn), 1.83 (2H, m, Hβ-Lys), 1.72 (8H, m, Hβ’ of both Orn, Hδ of both Orn, Hε-Lys), 1.42 (2H, m, Hγ-Lys); ^13^C-NMR: δ 174.44 (s, CO-Phe), 173.53, 173.29 (s, CO of both Orn), 170.09 (s, CO-Lys), 138.21 (s, Ar), 130.47 (d, Ar), 129.51 (d, Ar), 127.84 (d, Ar), 56.25 (d, Cα-Phe), 54.36 (d, Cα-Orn), 54.08 (d, Cα-Lys), 53.20 (d, Cα-Orn), 40.33 (t, Cδ of both Orn), 40.16 (t, Cε-Lys), 38.93 (t, Cβ-Phe), 32.06 (t, Cβ-Lys), 30.04, 29.70 (t, Cβ of both Orn), 29.00 (t, Cδ-Lys), 25.20, 24.92 (t, Cγ of both Orn), 22.78 (t, Cγ-Lys); FAB^+^MS *m/z* (% ra): 522 (24) [M + H]^+^, 307 (27), 289 (14), 154 (100), 136 (68), 107 (21), 89 (23), 77 (22); HRFAB^+^MS: observed 522.3398 [M + H]^+^, (calcd. for C_25_H_44_N_7_O_5_, 522.3404).

*Phe-Orn-Phe-Orn* (**4**). White solid; Mp 148-150 °C; R_f_ 0.26 (BuOH:AcOH:H_2_O, 2:4:1); [α] – 3.7 (c 0.62, MeOH); IR: 3,423, 3,073, 2,935, 1,676, 1,528, 1,199, 1,139, 839, 800, 715, 603 cm^-1^. ^1^H NMR: δ 7.37-7.12 (10H, m, Ar), 4.62 (1H, dd, *J* = 9.6, 5.2 Hz, Hα-Phe), 4.43 (2H, m, Hα of both Orn), 4.13 (1H, dd, *J* = 8.4, 6.0 Hz, Hα-Phe), 3.20, (1H, dd, *J* = 14.0, 8.4 Hz, Hβ-Phe), 3.13 (1H, dd, *J* = 14.0, 6.0 Hz, Hβ-Phe), 2.95 (6H, m, Hβ’ of both Phe, Hδ of both Orn), 1.99 (2H, m, Hβ of both Orn), 1.75 (6H, m, Hβ’ of both Orn, Hγ of both Orn); ^13^C-NMR: δ 174.61 (s, CO-Orn), 173.90 (s, CO-Phe), 173.07 (s, CO-Orn), 169.78 (s, CO-Phe), 138.35 and 135.60 (s, Ar), 130.62, 130.53, 130.19 and 129.71 (d each, C_2,6_ and C_3,5_ of both Phe), 128.88, 128.04 (d each, C_4_ of both Phe), 56.53, 55.69 (d each, Cα of both Phe), 53.76, 53.08 (d, Cα of both Orn), 40.27, 40.22 (t, Cδ of both Orn), 38.79, 38.72 (t, Cβ of both Phe), 30.24, 29.58 (t, Cβ of both Orn), 25.10, 24.72 (t, Cγ of both Orn); FAB^+^MS *m/z* (% ra): 522 (24) [M + H]^+^, 307 (27), 289 (14), 154 (100), 136 (68), 107 (21), 89 (23), 77 (22); HRFAB^+^MS: observed 522.3398 [M + H]^+^, (calcd. for C_29_H_43_N_6_O_5_, 522.3404).

*Lys-Phe-Phe-Orn* (**5**). White solid; Mp 114-116 °C; R_f_ 0.26 (BuOH:AcOH:H_2_O, 2:4:1); [α] + 6.6 (c 1.27, MeOH); IR: 3,422, 3,278, 3,074, 2,943, 1,677, 1,534, 1,197, 1,140, 839, 801, 713 cm^-1; 1^H-NMR: δ 7.36-7.16 (10H, m, Ar), 4.62 (1H, dd, *J* = 8.8, 5.2 Hz, Hα-Phe), 4.59 (1H, dd, *J* = 9.6, 5.2 Hz, Hα-Phe), 3.82 (1H, t, *J* = 6.8 Hz, Hα-Lys), 3.17 (1H, dd, *J* = 14.0, 5.2 Hz, Hβ-Phe), 3.07 (1H, dd, *J* = 14.0, 5.6 Hz, Hβ-Phe), 2.96 (6H, m, Hβ’ of both Phe, Hδ-Orn, Hε-Lys), 1.98 (1H, m, Hβ-Orn), 1.82 (1H, m, Hβ-Lys), 1.75 (3H, m, Hβ’-Orn, Hγ-Orn), 1.66 (2H, m, Hδ-Lys), 1.40 (2H,m, Hγ-Lys); ^13^C-NMR: δ 174.32 (s, CO-Orn), 173.44, 173.32 (s, CO of both Phe), 170.07 (s, CO-Lys), 138.30, 138.13 (2s, C_1_ of both Phe), 130.54, 130.18 (d, C_2,6_ of both Phe), 129.54 (d, C_3,5_ of both Phe), 127.87 (d, C_4_ of both Phe), 57.12, 56.38 (d, Cα of both Phe), 54.04 (d, Cα-Lys), 53.04 (d, Cα-Orn), 40.33 (t, Cδ-Orn, Cε-Lys), 38.86, 38.75 (t, Cβ of both Phe), 32.15 (t, Cβ-Lys), 29.74 (t, Cβ-Orn), 28.16 (t, Cδ-Lys), 25.20 (t, Cγ-Orn), 22.72 (t, Cγ-Lys); FAB^+^MS *m/z*: 555 (88) [M + H]^+^, 440 (13), 248 (10), 154 (76), 136 (68), 120 (95), 84 (100), 77 (32) [C_6_H_5_]^+^; HRFAB^+^MS: observed 555.3320 [M + H]^+^, (calcd. for C_29_H_43_N_6_O_5_, 555.3295).

*Orn-Phe-Lys-Val* (**6**). White solid; Mp 136-138 °C; R_f_ 0.30 (BuOH:AcOH:H_2_O, 2:4:1); [α] + 2.71 (c 1.7, MeOH); IR: 3,431, 3,293, 2,966, 1,675, 1,539, 1,436, 1,200, 1,135, 840, 803, 722, 449 cm^-1^; ^1^H- NMR: δ 7.32-7.17 (5H, m, Ar), 4.70 (1H, dd, *J* = 10.0, 4.8 Hz, Hα-Phe), 4.46 (1H, dd, *J* = 8.0, 6.0 Hz, Hα-Orn), 4.27 (1H, d, *J* = 6.0 Hz, Hα-Val), 3.92 (1H, t, *J* = 6.0 Hz, Hα-Lys), 3.20 (1H, dd, *J* = 14.4, 4.8 Hz, Hβ-Phe), 2.95 (5H, m, Hβ’-Phe, Hδ-Orn, Hε-Lys), 2.19 (1H, dh, *J* = 6.8, 5.6 Hz, Hβ-Val), 1.95 (3H, m, Hβ-Orn, Hβ-Lys), 1.91-1.65 (5H, m, Hβ’-Orn, Hδ-Orn, Hδ-Lys), 1.48 (2H, m, Hγ-Lys), 0.99 (3H, d, *J* = 6.8 Hz, Hγ-Val), 0.98 (3H, d, *J* = 6.8 Hz, Hγ’-Val);^13^C-NMR: δ 174.58 (s, CO-Val), 173.91 (s, CO-Phe), 173.40 (s, CO-Orn), 169.85 (s, CO-Lys), 138.09 (s, Ar), 130.20 (d, Ar), 129.60 (d, Ar), 127.94 (d, Ar), 59.43 (d, Cα-Val), 56.95 (d, Cα-Phe), 54.42 (d, Cα-Orn), 53.55 (d, Cα-Lys), 40.60 (t, Cδ-Orn), 40.22 (t, Cε-Lys), 38.86 (t, Cβ-Phe), 32.83 (t, Cβ-Lys), 31.70 (t, Cβ-Val), 29.64 (t, Cβ-Orn), 28.27 (t, Cδ-Lys), 23.85 (t, Cγ-Orn), 22.72 (t, Cγ-Lys), 19.79 (q, Cγ-Val), 18.65 (q, Cγ’-Val); FAB^+^MS *m/z* (% ra): 507 (100) [M + H]^+^, 307 (12), 246 (11), 154 (64), 136 (53), 120 (25), 107 (22), 89 (30), 77 (29), 70 (42), 63 (18); HRFAB^+^MS: observed 507.3288 [M + H]^+^, (calcd. for C_25_H_43_N_6_O_5_, 507.3295).

*Lys-Val-Pro-Orn* (**7**). Colorless syrup; R_f_ 0.13 (BuOH:AcOH:H_2_O, 2:4:1); [α] – 21.2 (c 0.43, MeOH); IR: 3,426, 3,080, 2,974, 1,680, 1,637, 1,541, 1,448, 1,202, 1,139, 841, 801, 722, 603 cm^-1^; ^1^H-NMR: δ 4.48-4.43 (3H, m, Hα-Pro, Hδ-Pro), 4.45 (1H, d, *J* = 8.0 Hz, Hα-Val), 4.42-4.35 (1H, m, Hα-Orn), 4.00 (1H, t, *J* = 6.8 Hz, Hα-Lys), 3.00-2.90 (4H, m, Hε-Orn, Hε-Lys), 2.28-2.18 (1H, m, Hβ-Pro), 2.15-2.91 (4H, m, Hβ-Val, Hβ-Pro, Hδ-Pro), 1.90-1.75 (6H, m, Hβ-Lys, Hβ-Orn, Hγ-Orn), 1.73-1.62 (2H, m, Hδ-Lys), 1.60-1.40 (2H, m, Hγ-Lys), 1.07 (3H, d, *J* = 6.8 Hz, Hγ-Val), 1.03 (3H, d, *J* = 6.8 Hz, Hγ’-Val); ^13^C-NMR: δ 174.03 (s, CO-Orn), 172.32 (s, CO-Pro), 170.16 (s, CO-Lys), 169.99 (s, CO-Val), 61.76 (t, Cδ-Pro), 61.40 (d, Cα-Pro), 58.74 (d, Cα-Val), 53.87 (d, Cα- Lys), 53.10 (d, Cα-Orn), 40.38 (t, Cδ-Orn), 40.21 (t, Cε-Lys), 32.24 (t, Cβ-Lys), 31.54 (t, Cβ-Val), 30.71 (t, Cβ-Pro), 29.73 (t, Cβ-Orn), 28.10 (t, Cδ-Lys), 26.20 (t, Cγ-Pro), 25.01 (t, Cγ-Orn), 22.21 (t, Cγ-Lys), 19.78 (q, Cγ-Val), 19.06 (q, Cγ-Val); FAB^+^MS *m/z* (% ra): 501 (37) [M + 2Na – H]^+^, 479 (100) [M + Na]^+^, 457 (43) [M + H]^+^, 364 (32), 347 (63), 319 (21), 230 (53); HRFAB^+^MS: observed 457.3215 [M + H]^+^, (calcd. for C_21_H_41_N_6_O_5_, 457.3138).

*Lys-Val-Phe-Pro* (**8**). White solid; Mp 161-163 °C; R_f_ 0.43 (BuOH:AcOH:H_2_O, 2:4:1); [α] – 16.98 (c 1.02, MeOH); IR: 3,421, 3,076, 2,969, 1,679, 1,637, 1,542, 1,452, 1,200, 1,140, 841, 801, 717, 604 cm^-1^; ^1^H-NMR: δ 7.35-7.18 (5H, m, Ar), 4.83 (1H, dd, *J* = 8.0, 5.6 Hz, Hα-Phe), 4.38 (1H, dd, *J* = 8.8, 4.4 Hz, Hα-Pro), 4.20 (1H, d, *J* = 7.6 Hz, Hα-Val), 3.96 (1H, t, *J* = 6.4 Hz, Hα-Lys), 3.85-3.65 and 3.52-3.41 (4H, m, Hδ-Pro and Hε-Lys), 3.11 (1H, dd, *J* = 13.6, 5.2 Hz, Hβ-Phe), 3.03-2.94 (3H, m, Hβ’-Phe), 2.93-2.87 (2H, m, Hε-Lys), 2.30-1.35 (2H, m, Hβ-Pro, Hβ-Val, Hβ-Lys, Hγ-Lys, Hδ-Lys), 0.92 (3H, d, J = 6.4 Hz, Hγ-Val), 0.87 (3H, d, J = 6.4 Hz, Hγ’-Val); ^13^C-NMR: δ 175.25 (s, CO-Val), 173.00 (s, CO-Pro), 171.82 (s, CO-Phe), 170.03 (s, CO-Lys), 138.19 (s, Ar), 130.60 (d, Ar), 129.56 (d, Ar), 127.86 (d, Ar), 73.07 (d, Cα-Pro), 64.92 (t, Cδ-Pro), 60.79 (d, Cα-Pro, Cα-Val), 54.20 (d, Cα-Phe), 54.00 (d, Cα-Lys), 40.38 (t, Cε-Lys), 38.49 (t, Cβ-Phe), 32.28 (t, Cβ-Lys), 31.97 (t, Cβ-Val), 30.30 (t, Cβ-Pro), 28.13 (t, Cδ-Lys), 26.03 (t, Cγ-Pro), 22.80 (t, Cγ-Lys), 19.85 (q, Cγ-Val), 19.00 (q, Cγ-Val); FAB^+^MS *m/z* (% ra): 490 (87) [M + H]^+^, 375 (11), 307 (10), 263 (15), 200 (16), 175 (49), 154 (83), 136 (76), 120 (50), 84 (100); HRFAB^+^MS: observed 490.3071 [M + H]^+^, (calcd. for C_25_H_40_N_5_O_5_, 490.3029).

*Phe-Orn-Val* (**9**). White solid; Mp 205-207 °C; R_f_ 0.46 (BuOH:AcOH:H_2_O, 2:4:1); [α] + 1.57 (c 0.69, MeOH); IR: 3,433, 3,077, 2,968, 1,681, 1,648, 1,537, 1,400, 1,201, 1,134, 838, 801, 592 cm^-1^; ^1^H- NMR: δ 7.36-7.25 (5H, m, Ar), 4.53 (1H, dd, *J* = 7.2, 6.0 Hz, Hα-Orn), 4.34 (1H, d, *J* = 5.2 Hz, Hα-Val), 4.20 (1H, dd, *J* = 8.0, 5.6 Hz, Hα-Phe), 3.27 (1H, dd, *J* = 14.0, 5.2 Hz, Hβ-Phe), 3.04 (1H, m, dd, *J* = 14.0, 8.0 Hz, Hβ’-Phe), 2.97 (1H, t, *J* = 7.2 Hz, Hδ-Orn), 2.23 (1H, dh, *J* = 6.8, 5.6 Hz, Hβ-Val), 1.90 (1H, m, Hβ-Orn), 1.77 (3H, m, Hβ’-Orn, Hγ-Orn), 1.01 (3H, d, *J* = 6.8 Hz, Hγ-Val), 0.99 (3H, d, *J* = 6.8 Hz, Hγ’-Val); ^13^C-NMR: δ 174.85 (s, CO-Val), 173.35 (s, CO-Orn), 169.77 (s, CO-Phe), 135.58 (s, Ar), 130.70 (d, Ar), 130.21 (d, Ar), 128.95 (d, Ar), 59.17 (d, Cα-Val), 55.57 (d, Cα-Phe), 53.92 (d, Cα-Orn), 40.30 (t, Cδ-Orn), 38.61 (t, Cβ-Phe), 31.74 (d, Cβ-Val), 30.36 (t, Cβ-Orn), 24.79 (t, Cγ-Orn), 19.79 (q, Cγ-Val), 18.41 (q, Cγ’-Val); FAB^+^MS *m/z* (% ra): 379 (80) [M + H]^+^, 262 (8) [M + H - C_5_H_10_NO_2_]^+^, 232 (8) [M + H - C_9_H_9_NO]^+^, 213 (8) [C_10_H_17_N_2_O_3_]^+^, 120 (100) [C_8_H_10_N]^+^, 115 (48) [C_5_H_9_NO_2_]^+^, 91 (13) [C_7_H_7_]^+^, 70 (42); HRFAB^+^MS: observed 379.2315 [M + H]^+^, (calcd. for C_19_H_31_N_4_O_4_, 379.2345).

## Figures and Tables

**Table 1:** Chemosuppression of *P. berghei* schizont cultures of peptides **1**-**9** after 16 h.

Compound (200 μM)	Chemosuppression of *P. berghei* schizont cultures (%)	IC_50_ (μM)
Ile-Lys-Phe-Orn (**1**)	83.97	32.35
Val-Lys-Phe-Orn (**2**)	85.85	10.00
Lys-Orn-Phe-Orn (**3**)	71.60	13.48
Phe-Orn-Phe-Orn (**4**)	85.94	3.31
Lys-Phe-Phe-Orn (**5**)	75.82	2.57
Orn-Phe-Lys-Val (**6**)	26.58	>200
Lys-Val-Pro-Orn (**7**)	27.39	>200
Lys-Val-Phe-Pro (**8**)	72.78	51.28
Phe-Orn-Val (**9**)	55.95	120.22
Chloroquine	100	0.06
